# Manoeuvring between anxiety and control: Patients’ experience of learning to live with diabetes: A lifeworld phenomenological study

**DOI:** 10.3402/qhw.v10.27147

**Published:** 2015-04-08

**Authors:** Karin Johansson, Sofia Almerud Österberg, Janeth Leksell, Mia Berglund

**Affiliations:** 1 Department of Health and Care Sciences, Faculty of Health and Life Science, Linnaeus University, Växjö, Sweden; 2 Department of Administration/Primary Care, Region Kronoberg County Council, Växjö, Sweden; 3 School of Health and Social Sciences, University Dalarna, Falun, Sweden; 4 Department of Medical Sciences, Uppsala University, Uppsala, Sweden; 5 School of Health and Education, University of Skövde, Skövde, Sweden

**Keywords:** Diabetes, existence, interviews, learning, lifeworld, phenomenology

## Abstract

Research shows that people with diabetes want their lives to proceed as normally as possible, but some patients experience difficulty in reaching their desired goals with treatment. The learning process is a complex phenomenon interwoven into every facet of life. Patients and healthcare providers often have different perspectives in care which gives different expectations on what the patients need to learn and cope with. The aim of this study, therefore, is to describe the experience of learning to live with diabetes. Interviews were conducted with 12 patients afflicted with type 1 or type 2 diabetes. The interviews were then analysed with reference to the reflective lifeworld research approach. The analysis shows that when the afflicted realize that their bodies undergo changes and that blood sugar levels are not always balanced as earlier in life, they can adjust to their new conditions early. The afflicted must take responsibility for balancing their blood sugar levels and incorporating the illness into their lives. Achieving such goals necessitates knowledge. The search for knowledge and sensitivity to changes are constant requirements for people with diabetes. Learning is driven by the tension caused by the need for and dependence on safe blood sugar control, the fear of losing such control, and the fear of future complications. The most important responsibilities for these patients are aspiring to understand their bodies as lived bodies, ensuring safety and security, and acquiring the knowledge essential to making conscious choices.

Diabetes is a long-term illness, which together with cancer, mental health problems, chronic respiratory disease, and musculoskeletal conditions are the non-communicable diseases that constitute the greatest disease burden in Europe (80%), causing 86% of deaths in the WHO European Region. Globally, the prevalence of diabetes is increasing in relation to growing problems with obesity and being overweight, physical inactivity, and socioeconomic disadvantage (WHO, 2013). Diabetes belongs to a group of chronic metabolic disorders characterized by elevated blood glucose levels; this condition affects about 4–6% of the population in Sweden (National Board of Health and Welfare, 2014; Thunander et al., 2008; Wild, Roglic, Green, Sicree, & King, 2004).

Diabetes considerably changes one’s life and gives rise to the need to learn how to live with the illness, which has been described in earlier Swedish studies (Andersson, Ekman, Lindblad, & Friberg, 2008; Berglund & Källerwald, 2012; Johansson, Ekebergh, & Dahlberg, 2009; Jutterstöm, 2013; Kneck, Klang, & Fagerberg, 2011). The area has not been studied internationally. A few studies focusing on young people with diabetes-related complications have been identified (Abu-Qamar & Wilson, 2012; Adams, 2010; Caro-Bautista, Martín-Santos, & Morales-Asencio, 2013; Seitz, Rosemann, Gensichen, & Huber, 2010; Spencer, Cooper, & Milton, 2012; Tan, Chen, Taylor, & Hegney, 2012). According to Merleau-Ponty (2002), the human being is a lived body that is biologically thinking, feeling, and acting at the same time. It is from the body the person understands himself/herself, others, and the world. Usually a person need not think of how the biological body functions but when the person is affected by a disease such as diabetes, a more considerate action is needed. Because the person, according to MP, has access to the world through his body, the disease changes and affects the person (Merleau-Ponty, 2002). The current work adheres to this understanding of the body as a lived body, A previous study shows that people who are newly diagnosed with diabetes desire for their lives to return to normal and for their new situation to be natural; these desires are related to the struggle over not being identified with the disease (Johansson et al., 2009). Patients and healthcare providers often have different perspectives in care. Toombs (1993) describes the patient perspective as illness and the medical perspective as disease; this gives different expectations on what the patients need to learn and cope with. Studies have shown that important aspects of treatment are achieving patient-centred interaction and sharing patients’ personal understanding of living with diabetes (Boström, Isaksson, Lundman, Graneheim, & Hörnsten, 2014; Hörnsten, Sandström, & Lundman, 2004).

Medical advances have made possible active self-care and control, as well as influence over treatment (Polonsky, 2002). Experience of balancing biomarkers in relation to the quality of life in the long term has been described by people with type 2 diabetes in the study (Frost, Garside, Cooper, & Britten, 2014). The outcome of treatment and patient education is measured in terms of HbA1c, a blood sample that is strongly correlated with the development of complications (UK Prospective Diabetes Study Group [UKPDS], 1998). A sufficient level of HbA1c is a goal for the healthcare system and often also is in line with what the person living with diabetes considers as good control of the disease (Adolfsson, Smide, Rosenblad, & Wikblad, 2009; Ellis, Speroff, Dittus, Pichert, & Elasy, 2004; Gary, Peyrot, & Brancati, 2003; Norris, Engelgau, & Narayan, 2001). Despite the importance of this measure, however, studies (Eeg-Olofsson, Cederholm, Nilsson, Gudbjörnsdóttir, & Eliasson, 2007) and national quality registers for diabetes (National Diabetes Register, 2013) have shown that the majority of afflicted individuals do not reach their expected HbA1c goals.

For various reasons, many persons with diabetes experience difficulty in applying necessary lifestyle changes and adhering to treatment recommendations (Söderberg & Karlsson, 2005). A problem can be that patient education often is organized after a pre-planned programme that defines patient needs in accordance with the identification of carers and more strongly emphasizes the medical component rather than the existential element of the disease (Dahlberg & Segersten, 2013). Previous studies on patient education regarding diabetes are typically designed to evaluate the efficacy of training efforts, as measured by various metrics (HbA1c) that provide an outside perspective on the effects of the disease (Swedish Council on Technology Assessment in Health Care [SBU], 2009). Evaluation based on patients’ perspectives is seldom performed (Steinbekk, Rygg, Lisulo, Rise, & Fretheim, 2012).

Supporting patients’ learning processes from an inside perspective and incorporating the disease into their lives necessitates knowledge about how the patients should be educated and how carers can satisfy their need for learning (Friberg & Hansson-Scherman, 2005). The importance of understanding patients’ learning processes is insufficiently emphasized in the literature. Learning to live with diabetes from persons with a diabetes perspective is described by Kneck et al. (2011) as a search for knowledge that facilitates understanding of bodily changes. Hörnsten et al. (2004) elucidate the manner by which an individual’s understanding of diabetes develops and indicate that understanding enables the afflicted to assign the disease a place in daily life. This viewpoint aligns with that of Berglund (Berglund, 2011, 2014; Berglund & Källerwald, 2012), who use lifeworld theory (Heidegger, 2008; Husserl, 1975; Merleau-Ponty, 2002) as a basis in studying the learning process that drives living with a long-term illness. The author defines the learning process as a complex phenomenon interwoven into every facet of life. During this process, learning results are understood as when a patient familiarizes himself/herself with the disease, as well as incorporates and assigns it a space in his/her life. The results of learning are knowledge, new understanding, and a capability to think and act, that is, to perform self-management. In that way a disease can challenge a person to get a new understanding of himself and his situation Berglund, 2011, 2014). The current work subscribes to this definition of learning, and its aim is to describe what it means to learn to live with diabetes. This understanding and insight can, with advantage, be used at individual as well as group-based interventions.

## Methods

Learning to live with diabetes was explored and illuminated by the reflective lifeworld research approach (RLR), which is based on phenomenological epistemology, as described by Dahlberg, Dahlberg, and Nyström (2008). In the RLR approach, openness is used as a guiding tool for the methodological work. This entails the researchers’ understanding, and in particular their pre-understanding, being bridled in relation to the phenomenon studied (Dahlberg & Dahlberg, 2003).

### Participants and data collection

To explore people’s experience with the disease, interviews were conducted (Dahlberg, Dahlberg & Nyström, 2008) with participants who were recruited from four care units (one specialist clinic and three primary care units) in South Sweden. Each unit recruited three Swedish-speaking persons of various sexes, ages, and experiences (i.e., time spent) with the disease and treatment. The participants, who were equipped with different forms of patient education (represented in the region, solution-focusing method (De Jong & Berg, 2008), learning and coping (Læring og mestring, mestring.no, 2015), and traditional), were five men and seven women between the ages of 76 and 45 and who had been living with the disease for 2–46 years. Three persons had type 1 and nine persons had type 2 diabetes, with an age at disease onset varying from 13 to 74 years. The participants chose the location of the interviews (five chose to be interviewed at home and seven decided to have their interviews at a regular care unit), which lasted between 45 and 75 min. The interviews were carried out in conversational form and initiated with opened questions regarding how the respondents experienced falling ill and how they learned to live with the disease. Follow-up questions (e.g., tell me more, in what way, how did you experience it) were raised to gain deeper insight into the respondents’ experiences.

### Data analysis

The method adopted in this work can be described as a dialectic process (Dahlber, Dahlberg & Nyström, 2008). The initial analysis focused on the situation as a whole, followed by an examination of the components and concluded with a reconstruction of the whole to understand the essence of the phenomenon being studied. Specifically, the interview transcripts (regarded as the whole text) were analysed in a general manner, after which units of meaning (a word, a sentence, or a longer section of text) were identified. The meanings were scrutinized and compared with the background of the whole. The next step was to build clusters that are identical to the groups of meanings. After all the units of meanings in the data were analysed and clustered into groups, the essence was recreated. According to Dahlberg and Dahlberg (2003), the essence of a text is an abstraction and synthesis of the structure of meanings that converts a phenomenon into the actual phenomenon and nothing else. The essence can therefore be understood as a new whole.

The analysis was initiated by listening to the interviews for familiarization with the responses of the interviewees. After similarities, differences, and patterns of meaning were identified, the text was examined for what was said, how it was said, and what meaning was attached to what was said (e.g., the manner by which a participant described the experience of learning). Observing the similarities and differences in the material revealed a pattern of experiences and meanings. The subjective lifeworld perspective expressed in the interviews was transformed into a professional and scientific description, with focus on the studied phenomenon.

During the course of the work, the initially identified pattern changed character, thereby compelling us to shift attention between the whole and its parts before the analysis finally resulted in a definitive description of the essence. The essential structure was further described on the basis of its constituents. With the constituents, the meanings carried by the phenomenon in question could be described on a more concrete level. The Results section presents the essential structure, followed by its constituents. The essence describes the general structure of the phenomenon, and the constituents elucidate its aspects and variations. Sample interview transcripts are included to illustrate the described meanings.

### Ethical considerations

Approval for the study was granted by the Regional Ethics Committee of Linköping (Dnr. 2012/222-32). Field officers approved participation. The participants were presented with oral and written information about the aim of the study and were asked to provide written consent.

## Results

To learn to live with diabetes means realizing that the body no longer functions in a biological way that has been taken for granted. Trusting the body to balance blood sugar levels on its own is no longer possible. Food, activities, and moods influence blood sugar levels in a way that should be mastered, which requires a constant search for knowledge. Learning means taking control and protecting the body from harm in the short- and long-terms. Learning means integrating the disease as change biological body in the understanding of one’s self as a lived body with diabetes as a disease and as an illness. This results in enabling oneself to live to the fullest under new conditions. The learning process demands an understanding that individuals must act on and take responsibility for—a responsibility that is imposed and constantly present. Learning involves handling the changes that occur over time and in available knowledge about the illness and its treatment. Furthermore, learning is driven by the need for safe blood sugar control, the fear of losing such control, and the fear of dependence on checks and future complications. It pertains to the ability to immediately read signals and apply knowledge, with the aim of adjusting blood sugar levels. In so doing, a diabetic controls such levels, thereby fostering the courage to accept the new challenges presented by the illness.

The phenomenon under study is further enlightened by its four constituents: Handling changes in the body or in treatment advice, incorporating the illness and its treatment into daily life, manoeuvring between fear and control, and taking responsibility for acquiring and applying new knowledge.

### Handling changes in the body or in treatment advice

As previously stated, learning to live with diabetes means learning to handle changes in the body and in knowledge about the illness and its treatment. This means becoming proficient in independently dealing with changes and other people’s attitudes towards the illness. Such learning pertains to developing the ability to recognize the signs of changes and an understanding that the body no longer controls blood sugar levels in a way previously taken for granted, as well as an understanding that the body continues to change and react in different ways. These changes coincide with natural variations in life, with the fact that diabetes influences and changes the body and with the possibility that more illnesses can consequently occur.

Bodily signals for high and low blood sugar levels vary from person to person and over time. In the initial stage of the illness, clear signals can be detected but may be difficult to connect with a high blood sugar level. One of the interviewees describes these difficulties in the following manner:

I had felt a bit strange, lost my balance sometimes, thought that I had a sluggish brain, felt like syrup. What I could figure out before … see what was wrong I could no longer do … I simply felt stupid, a dunce.

Learning also means establishing an understanding that the body’s regulation of blood sugar levels changes not only upon disease onset, but continually over time and across different situations. This knowledge can be difficult to accept until bodily changes are experienced, and the connection between the changes and the illness is realized. Bodily signals are influenced by various factors and can be relatively easy to interpret in certain situations. One of the female respondents states that “if you are physically tired, it’s harder to say whether it’s the illness or fatigue. When you are rested, it’s easier.” Learning also means handling the natural hormonal changes caused by puberty or menopause, which is described as “the illness that lives its own life.” Blood sugar levels are also influenced by emotions, such as happiness, fear, stress, and sadness. Another female respondent describes the situation as follows: “I was very sad and since feelings influence the blood sugar level, it rose.” Awareness of the factors that cause changes in blood sugar levels enables individuals to learn to live with diabetes. Experiences, observations, and reflections shed light on how one’s body reacts to different situations. Other necessary characteristics, therefore, are curiosity and the will to critically scrutinize one’s way of living. A male respondent shares that for a given period, he carefully monitored how different dishes changed his blood sugar levels.

So I made a super fine Excel programme … I know exactly what I can and can’t eat. I bought food, test cooked, and ate and after 1 hour, after 2 hours, after 4 hours, I made tables of everything, so I can say I have super control.

Learning also means daring to trust and test new knowledge about the illness and its treatment. It means risking abandoning old knowledge that has been evaluated as “true” and has become part of one’s way of life. Such a move can translate to a shift in habitual treatment, for example, from diet and exercise to tablets and insulin treatments. Additionally, learning pertains to having the courage to trust new techniques, through which persons with diabetes can actively participate in development. A male interviewee who changed his treatment from insulin injections several times a day to using an insulin pump describes how this shift affected his life: “… and feel well like this and sort of feel the usefulness of the pump, for as if by magic it really turned my life around.” Courage, will, and curiosity are necessary to modifying a treatment that one is used to and learning to live with a new technique.

Medical developments have changed the way we use technical aids. Early innovations enabled only uncertain measurement with urine strips. Today, the possibilities available to persons with diabetes in terms of monitoring blood sugar levels are much greater. For the afflicted, these possibilities also translate to an increase in power, which is described by one of the respondents in the following manner: “It was very exciting and very comforting for it gave you the power to control your own disease so that you knew that the situation was good.” By testing, conducting trials, and reflecting over bodily and knowledge-related changes, one can successfully live with diabetes.

### Incorporating the illness and its treatment into daily life

Learning means incorporating the illness and its treatment into daily life and directing the treatment so that it corresponds with changes in the body’s needs. Understanding such changes provides insight into available possibilities and the different circumstances that influence the illness. It also sheds light on what has happened to a diabetic, providing him/her the realization that although the situation is unchangeable, influencing it is possible. Learning means coming to terms with one’s new conditions. It affords persons with diabetes the motivation to carry out necessary changes.

Learning to live with diabetes pertains to a realization that can be described as the initiation of the learning process, which leads to the incorporation of the illness and its treatment as a natural part of everyday life. A person with diabetes receives information on being a diabetic in different ways to realize, accept, and incorporate it into his/her life. Being diagnosed with diabetes can be viewed as a catastrophe that is difficult to come to terms with. One of the respondents describes the experience thus:

I had an infection, gastric influenza or something like that, but then I fell asleep for real and became unconscious at home so they had to go to the emergency ward … then I woke up and there was this nurse sitting beside me and then I said where am I? You’re in hospital she said and you have diabetes so you will have to take insulin injections for the rest of your life. Never in a thousand years I said then.

Another interviewee shares the following experience:

To be told I had the illness created anger.… Had to be angry for a while but then the anger disappeared and I could get on with my life with the illness … The illness must not govern my life but be a part of it.

After some time, the illness was given a place in the interviewees’ lives: “The illness is a rather OK mate to live with. It keeps itself rather quiet and is just there, sometimes you have to take it into consideration but that’s OK.”

Conditions relatively change because the illness is akin to a rucksack that you carry on your back. For some, the illness is expected given that it is hereditary or is a natural progression caused by old age. “What do you expect when you’re 76, not much to think, only had to accept it.”

Even though the illness is not a secret, those afflicted do not want to openly display its effects. One of the participants states that “you feel more human if you go aside to check the blood sugar level.” They do not want the illness to attract attention; thus, they discreetly monitor their blood sugar levels. “If you say, you feel a little strange you get 10 eyes on you so it is better to sneak away and check.”

The assimilation of the illness and its treatment is facilitated by new knowledge, such as carbohydrate count and new aids, including insulin pumps. These advances are recognized as facilitative of adaptation to treatment and different situations. Adaptation can be regarded as part of assimilation, but this process does not automatically happen. Carbohydrate count and insulin pumps are aids that require focused handling. When pump treatment works, it affords persons with diabetes a sense of freedom, enabling the afflicted to be more easily attuned to their bodies’ needs. One of the participants explains the experience as follows: “I think we who have pumps are to some degree well. We live like healthy persons anyway.” Learning means incorporating knowledge, with the aim of adeptly handling aids, and consequently, minimizing the influence of the illness on one’s life.

### Manoeuvring between fear and control

Learning to live with diabetes refers to the ability to manoeuvre between the need for control over the illness and the fear of losing such control. The degree of fear varies with experience of the illness and with treatment. The possibility of control through technical aids creates security but can also manifest as a compulsive act.

Learning even means acknowledging and understanding fear and creating strategies for living even as fear is constantly present. Distress over complications and treatments constitute the motivation to apply necessary changes. Facts about the illness and the risk of consequences can also appear frightening; it gives people the sense that the situation will be difficult to handle. One of the respondents describes the fear that he experienced when he was diagnosed with diabetes:

My first thought is blind and amputated/ and then I went online and read about it, which I shouldn’t have done, because then I saw the bit about cardiac infarction. Then I noticed that you have twice as large a risk to have a coronary as a diabetic .… Yes, added to that in my family both mother and father have had coronaries, so that didn‘t make it any better, no, so it was a bit hard after a while, a bit about how to handle it.

Even the fear of losing control over blood sugar levels, feeling insulin effects, and becoming unconscious will have to be managed. In such a situation, the individual is learning to manoeuvre between the fear of losing control and becoming fixated on monitoring blood sugar and taking safe medicine. Additional blood sugar checks when a person is alone or has completed physical exercises can, for example, create security, thereby enabling that individual to safely relax and rest. “If I lie alone or have been about a lot during the day it happens that I set the alarm in the middle of the night to check the blood sugar level, then I sleep on.”

Learning to live with diabetes means mastering anxiety over deviating blood sugar levels—a worry that leads to concern for safety and can mean certain limitations on life. Checking routines before activities are described thus:

Before walking, driving a car a considerable distance, I check the blood sugar level I don’t want to expose myself to the risk of having a too low or too high blood sugar level. Both for my own and others’ safety.

These checks sometimes cause a person to abstain from an activity but also afford the afflicted possibilities for safely engaging in different occupations through medication and diet that are designed to address deviating blood sugar levels. Mastery over one’s anxiety is supported by knowledge gained through theory and experience. These complement each other and make learning more useful. The afflicted acquire good insight into what happens with blood sugar levels in different situations and the strategies that they can apply to influence such levels. This insight, in turn, makes room for activities and enables conscious choice. As described by a male respondent:

I can take one little slice of cake, but then it is noticeable if I have been to a party, because I take blood sugar test every 3 days, if I have been to a party it can be seen immediately … and then it takes a few days before I’m down again.

The worry that one experiences can always be alleviated with a blood sugar check. In such a situation, an individual finds himself/herself conflicted as to whether to trust what his/her body feels or what the technology reveals. Despite the benefits of monitoring, however, it can become a compulsive act: “Actually, I wouldn’t have had to check because I feel well.” Checks on blood sugar levels are carried out only because a meter is nearby or because measuring at a particular time has become a habit. Learning means not only understanding the possibilities that technical aids provide, but also learning to use them in accordance with one’s needs, instead of being enmeshed in compulsive acts.

Without the meter you would be lost, yes you would, especially since I’ve had diabetes for such a long time and don’t feel in good time that I have rather low blood sugar and still perhaps can appear to be rather clearheaded, keep a straight face and so. I think the meter is simply incredibly good, it really is.

To manoeuvre between fear and control means looking forward and avoiding being preoccupied with feelings of guilt over what could have been done differently earlier in life. Guilt is balanced against happiness over the absence of injuries due to lack of blood sugar control. As expressed by a female respondent: “I am free from side effects so you can see that the body can endure rather a lot.”

Learning to live with diabetes involves maintaining balance between the security offered by what one is used to and the possibilities presented by new technology, which can cause fear of losing control. The realization that serious events can happen in the short- and long-terms fuels the will to change one’s habits.

### Taking responsibility for acquiring and applying new knowledge

Learning to live with diabetes manifests itself as a will and the ability to acquire and implement knowledge, so that life as a diabetic becomes manageable. Knowledge is obtained from theories discussed in books, journals, and research reports and from information provided by health care personnel and other persons with diabetes. Learning is achieved through reading, testing, and evaluating different kinds of information. This approach develops knowledge in the form of strategies that support one’s actions, and such development demands sensitivity to bodily signals and the resolve to learn new things.

Learning also means tactfully using knowledge in relation to the life one lives. It revolves around taking responsibility both for the illness and for life’s short- and long-term opportunities.

As explained early in the article, learning pertains to comprehending how one’s body behaves; such understanding is supported by blood sugar checks in different situations, wherein an individual develops sensitivity to how blood sugar feels in the body and subsequently reflects over the factors that may have affected a reading. This testing and analysis verify or invalidate the evaluations that an individual may form in relation to the sensations that blood sugar levels generate. In addition, learning points to developing sensitivity to how a body changed by the illness signals blood sugar levels. One of the participants explains this development in the following manner: “… good blood sugar, super good, it feels as if you’re dancing in a different way.” Poor blood sugar levels are experienced thus: “… high, then your steps get heavy.” Another respondent describes the variation as follows:

You notice the difference, for it takes much longer, it sort of takes time to reach a decision, it doesn’t connect when you’re low, and if you’re too high then I get very …, I get tired, and I don’t feel well … my head feels that now I am tired and it feels sluggish.

One of the participants, however, does not experience variations in blood sugar levels: “No, damn, not a bit, no, it is just that strip, then you see that it is high.” Learning means fine-tuning one’s sensitivity in order to effectively interpret body signals. Sometimes, this fine-tuning is driven by another change. For example, one of the persons with diabetes relates that “vaccination gave very vacillating blood sugar.” To take responsibility, a watchfulness and the ability to critically evaluate signals are required. The vaccination prompted the woman to look into the matter further when she was dissatisfied with the initially provided information that “it was just diabetes.” She appreciated another health care worker’s recommendation that “a dermatologist will have to look at these red dots, I don’t know if it has anything to do with diabetes.”

Learning means a constant wish to acquire new knowledge, a will to understand in a new way, and a refusal to accept not being fully informed. In describing his way of learning, one of the respondents states that he was “curious to learn a lot about it so therefore I read very much at the beginning, I didn’t think now I have read a book so now I know, but I read everything that was new, searched, and looked.” Learning means being attentive to and collecting knowledge from different media.

Individuals can search for knowledge through established channels, such as books and brochures, as well as through health personnel and mass media. Knowledge can also be obtained from websites, applications, and chat clubs the world over. Diabetes journals are a good source of information that is presented through scientific reports and advertisements about novel technologies. Learning also means acquiring knowledge from one’s own experiences through reflection over a reading. For example, “if I measure a high blood sugar level, then I reflect a little over what I have eaten and what can have caused it.”

Learning to take responsibility for the treatment entails forming an understanding of how blood sugar levels are influenced. One’s own experiences can be compared with those of others to increase understanding. For this purpose, chat rooms on the Internet are good platforms for exchanging experiences. As one of the male participants relates:

Clubs on the net 5.0-club and 4.6-club … groups where someone who had diabetes told about his diabetes … they also say what they ate, 100 grams of that food and then I got that blood sugar reading, in that way you share.

Participation in a chat club can also serve as inspiration for mapping, in which an individual traces and monitors how his/her body works and uses the knowledge gained from this activity as support for making decisions.

If you want high or low blood sugar levels, then you chose the lowest and start from there. How can I get even lower and still feel that it’s good, until you find this low is where I want to be, but I don’t want to eat gravel, consequently I go up a bit and land somewhere and am satisfied, on the other hand I know that if I eat gravel it will be lower but I don’t want that.

A new technique can develop into a natural habit, thereby diminishing the feeling of being ill. One of the female respondents describes the development as follows:

Then, because you have learnt more, to exercise, eat and then be a bit active if you can … then you don’t take it so seriously but try instead just to be, this is me and not an illness, then emotionally you simply don’t feel ill.

Finally, learning also means knowledge that the ultimate responsibility and choices fall on the person with diabetes themselves. The afflicted carry this obligation, decide on the pace with which adjustment proceeds, and hold the possibility of influencing results in their hands.

## Discussion

The results of this study indicate how a person with diabetes focuses on learning about the body and the self in a new, and at the same time, constantly changing situation. As a result, after acknowledging the disease, two themes emerged as particularly important for those learning to live with diabetes: taking responsibility for acquiring knowledge for the purpose of incorporating the disease into daily life; and achieving balance between fear and control. According to Merealy-Ponty’s (2002) thoughts about the lived body, where thinking, feeling, acting, and biological functions not can be separated from each other, it is important that the illness and its treatment is given a prominent place for a period of time. In this learning process the illness becomes a natural part of the lived body through which life is lived. Earlier studies have given rise to speculations that people afflicted with diabetes have been forced to accept the disease as a prerequisite to moving on with their lives (Johansson et al., 2009). The results of the present study reveal a similar situation. Berglund (2011) describes the risk associated with forcing acceptance and recommends a dialogue to challenge the afflicted into reconciliation with the actual situation. Reconciliation is described as a turning point for change, as confirmed by Hörnsten et al. (2004). Berglund and Källerwald (2012) explain that genuine learning occurs when patients shift from ignoring their situation to allowing the illness to be a part of their lives. In the current research, personal responsibility manifests itself as “it is me who does it” and in the act of acquiring knowledge to understand how a diabetic’s body reacts. This understanding is created by testing and reflecting over results. The difference between a person with diabetes taking charge of his/her situation and not anyone in general is described by Berglund (2011) as an expression of personal responsibility. Kneck et al. (2011) define it as understanding the new capacity of the self and body in a new life situation. Heggdal (2013) terms it “embodied knowledge”—“a fundamental process for the development of personal knowledge about one’s own body, coping skill, health, and wellbeing.” Berglund (2011) describes learning as complex and as entailing more than receiving and following advice; it revolves around continually changing as a person. Taking personal responsibility necessitates genuine learning, which requires support, as also revealed in the present work.

The results also show how the participants have learned to balance between fear and control. Control over blood sugar levels provides an opportunity to learn how a body that is changed by diabetes reacts in different situations; such learning, in turn, affords persons with diabetes a feeling of security. This finding is confirmed by Kneck et al. (2011) and Cox, Gonder-Fredrick, Julian, and Clark (1994). The present study also shows increased personal power and self-confidence, which stem from the possibilities with which blood sugar levels can be measured. The drawback to measurement, nonetheless, is the fear of dependence on technology and diminishing trust in one’s own interpretations of bodily signals. The aim for persons with diabetes, therefore, is to regain and maintain balance in life and in measurements of blood sugar levels. Lipworth, Hooker, and Carter (2011) point out that balance can be viewed as an ideal state and as a process underlain by internal and external perspectives. Balance is a powerful, culturally recognized concept related to living the best possible life, with profound effects on health-related circumstances. Our results highlight the need for various means of support for achieving balance; that is, support that enables good choice of activities, food, and treatment and the exploration of limitations in the form of failed regulation of blood sugar levels. To accomplish balance in life also sometimes means opting for less favourable choices to achieve good quality of life. The participants in the present study state that they sometimes chose less favourable choices to improve adaptation to social life and activities. This situation corresponds with the theory that sometimes the disease captures all the attention and at other times, it stays in the background. This knowledge can be viewed similar to that described by Paterson’s (2001) shifting perspectives model and Heggdal’s (2013) body knowledging phases.

The result of the study highlights the importance of making the disease one’s own—a strategy that is important knowledge in patient meetings. To support an active searching for knowledge and new solutions it is important that the care personnel is attentive to the need for support which means that the care personnel with sensitivity understands the world and understanding of the learner, that is, pedagogical tact (Ekebergh, 2004; Van Mannen & Li, 2002). They should exercise pedagogical tact or diplomacy in which meetings should not be underlain by stringent rules (Ekebergh, 2004; Van Mannen & Li, 2002). Bullington (2007) emphasizes the cruciality of encouraging a patient’s active hand in managing the disease and care personnel’s stepping back in instances wherein only supplementary assistance is required. Similarly, Andersen and Funnell (2010) explain that empowerment begins when health care providers acknowledge that patients are in control of their daily diabetes care. The afflicted balance the fear of losing control and being excessively preoccupied with the need for blood sugar measurement. The results of the present research raise questions about how to design support to help persons with diabetes enunciate fear. The manner by which self-confidence is built and by which security in handling the body is guaranteed is supported, thereby enabling the disease to become a natural part of life.

The study was conducted in accordance with the RLR approach, and the respondents were sincere in their participation, as evidenced by the numerous contributions that they made regarding how patients can live with diabetes. Throughout the study, the researchers sought to maintain an open attitude and regularly reflect on their pre-understanding (Husserl, 1975). In the present study we have strived to bridle our pre-understanding (Dahlberg et al., 2008). The bridle has meant striving towards a scientific and reflective position where the researchers slow down and are conscious of their understanding of the phenomenon, as far as possible. Here critical questions have been asked regarding the result, for example: Is this meaning in the material? Has the analysis been influenced by a pre-understanding? KJ and JL are diabetic nurses, whereas the other authors are not, and have been able to be more critically open. All authors have participated in discussions to reach a deeper insight and understanding of the unique meaning and significance of the patients’ experiences, even if KJ supported by MB has been the driving person in the process. In this way we mean that the phenomenological approach has allowed the description of a richness and varied meaning of phenomena to come forward. According to (Dahlberg et al., 2008) the essential meaning of the phenomenon is an abstraction that never can be understood as universal, but can, with care, be generalized and transferred to groups within similar context.

## Conclusion

This study describes the experience of learning to live with diabetes from patients’ perspective—the primary factors identified are “taking responsibility for acquiring knowledge for the purpose of incorporating the disease into daily life and achieving balance between fear and control.”

What emerges as important aspects in life with diabetes are learning to understand that the lived body has been changed by the illness and the courage to trust one’s body’s subtle signals has become important. Other goals are to build a safe and secure life with diabetes disease and to obtain knowledge that enables a conscious choice. The result contributes to the possibility of understanding the patient from his lifeworld where access to life has been changed by the illness. The knowledge that this article gives is the understanding of how the carers meet every individual patient and how to be able to give support to the afflicted to meet his fear and be able to balance between anxiety and control.

Future work will describe what a person suffering from diabetes experiences while supporting their learning to live their life with the disease.
